# Supplementary material for: Clinical development success rates and social value of pediatric Phase 1 trials in oncology
Source: PLoS One. 2020 Jun 24;15(6):e0234911. doi: 10.1371/journal.pone.0234911 (PMC7313751; doi:10.1371/journal.pone.0234911)
Supplement: S1 Table — (DOCX) [file pone.0234911.s001.docx]

**S1 Table. Trials that achieved approval.**

|  | | **FDA** | | | **EMA** | | |
| --- | --- | --- | --- | --- | --- | --- | --- |
| **First author and year of publication** | **Drug** | **Initially approved pediatric indication (year of first pediatric approval)** | **Time lag between publishing and changing label** | **Source(s) link** | **Initially approved pediatric indication (year of first pediatric approval)** | **Time lag between publishing and changing label** | **Source link** |
| Jeha 2004 [1] | Clofarabine | Pediatric patients 1 to 21 years old with relapsed or refractory ALL after at least two prior regimens (2004) | 0 years (same year as Phase 1 publication) | <https://www.accessdata.fda.gov/drugsatfda_docs/label/2004/021673lbl.pdf> | Children and adults up to 21 years of age who have ALL which has not responded to or has relapsed after at least two other treatments (2006) | 2 years | <https://www.ema.europa.eu/en/documents/overview/evoltra-epar-summary-public_lt.pdf> |
| Kurtzberg 2005 [2] | Nelarabine (506U78) | Patients with T-ALL and T-LBL whose disease has not responded to or has relapsed following treatment with at least two chemotherapy regimens (2005) | 0 years (same year as Phase 1 publication) | <https://www.accessdata.fda.gov/drugsatfda_docs/label/2009/021877s001lbl.pdf>  <https://www.accessdata.fda.gov/drugsatfda_docs/label/2005/021877lbl.pdf> | Patients with T-ALL and T-LBL whose disease has not responded to or has  relapsed following treatment with at least two chemotherapy regimens (2007) | 2 years | <https://www.ema.europa.eu/en/documents/product-information/atriance-epar-product-information_en.pdf> |
| Pollack 2007 [3] | Imatinib | Newly diagnosed pediatric patients with Ph+ CML in chronic phase Pediatric patients with newly diagnosed Ph+ ALL in combination with chemotherapy (2007) | 0 years (same year as Phase 1 publication) | <https://www.accessdata.fda.gov/drugsatfda_docs/label/2007/021588s020lbl.pdf> | Pediatric patients with newly diagnosed Ph+ CML for whom bone marrow transplantation is not considered as the first line of treatment pediatric patients with Ph+ CML in chronic phase after failure of interferon-alpha therapy, or in accelerated phase or blast crisis pediatric patients with newly diagnosed Ph+ ALL integrated with chemotherapy (approved for pediatric population, exact date unclear) | N/A | <https://www.ema.europa.eu/en/documents/smop/chmp-post-authorisation-summary-positive-opinion-glivec_en.pdf> |
| Ladenstein 2013 [4] | Ch14.18 CHO (APN311, dinutuximab beta) | Pediatric patients with high-risk neuroblastoma who achieve at least a partial response to prior first-line multiagent, multimodality therapy (2015) | 2 years | <https://www.accessdata.fda.gov/drugsatfda_docs/label/2015/125516s000lbl.pdf> | High-risk neuroblastoma in patients aged 12 months and above, who have previously received induction chemotherapy and achieved at least a partial response, followed by myeloablative therapy and stem cell transplantation patients with history of relapsed or refractory neuroblastoma, with or without residual disease (2015) | 2 years | <https://www.ema.europa.eu/en/documents/product-information/unituxin-epar-product-information_en.pdf> |
| Zwaan 2013 [5] | Dasatinib | Pediatric patients with Ph+ CML in chronic phase (2017) | 4 years | <https://www.accessdata.fda.gov/drugsatfda_docs/label/2017/021986s020lbl.pdf> | Newly diagnosed Ph+ CML in chronic phase resistant or  intolerant to prior therapy including imatinib (2015) | 5 years | <https://www.ema.europa.eu/en/documents/variation-report/sprycel-h-c-000709-x-0056-g-epar-assessment-report-variation_en.pdf> |
| Fouladi 2007 [6] | Everolimus | Pediatric patients with tuberous sclerosis complex who have subependymal giant cell astrocytoma that requires therapeutic intervention but cannot be curatively resected (2012) | 5 years | <https://www.accessdata.fda.gov/drugsatfda_docs/label/2012/022334s018lbl.pdf> | N/A | N/A | <https://www.ema.europa.eu/en/documents/product-information/afinitor-epar-product-information_en.pdf> |
| Champagne 2004 [7] | Imatinib Mesylate (STI571) | Newly diagnosed pediatric patients with Ph+ CML in chronic phase pediatric patients with newly diagnosed Ph+ ALL in combination with chemotherapy (2007) | 3 years | <https://www.accessdata.fda.gov/drugsatfda_docs/label/2007/021588s020lbl.pdf> | Pediatric patients with newly diagnosed Ph+ CML for whom bone marrow transplantation is not considered as the first line of treatment pediatric patients with Ph+ CML in chronic phase after failure of interferon-alpha therapy, or in accelerated phase or blast crisis pediatric patients with newly diagnosed Ph+ ALL integrated with chemotherapy (approved for pediatric population, exact date unclear) | N/A | <https://www.ema.europa.eu/en/documents/smop/chmp-post-authorisation-summary-positive-opinion-glivec_en.pdf> |

N/A – Not Applicable, ALL – Acute Lymphoblastic Leukemia, AML – Acute Myeloid Leukemia, Ch14.18/CHO – human-mouse chimeric monoclonal anti-disialoganglioside GD2 antibody ch14.18 produced in Chinese hamster ovary cells, CLL – Chronic Lymphocytic Leukemia, CML – Chronic Myeloid Leukemia, CML-BC – Chronic Myeloid Leukemia Blast Crisis, EMA – European Medicines Agency, EU – European Union, FDA – Food and Drug Administration, LBL – Lymphoblastic Lymphoma, LPD – Lymphoproliferative Disorder, N/A – not applicable, NHL – non Hodgkins Lymphoma, Ph+ – Philadelphia chromosome positive, PLL – Prolymphocytic Leukemia

**Referances:**

1. Jeha S, Gandhi V, Chan K, McDonald L, Ramirez I, Madden R, et al. Clofarabine, a novel nucleoside analog, is active in pediatric patients with advanced leukemia. Blood. 2003 Oct 2;103(3):784–9.

2. Kurtzberg J, Ernst TJ, Keating MJ, Gandhi V, Hodge JP, Kisor DF, et al. Phase I Study of 506U78 Administered on a Consecutive 5-Day Schedule in Children and Adults With Refractory Hematologic Malignancies. J Clin Oncol. 2005 May 20;23(15):3396–403.

3. Pollack IF, Jakacki RI, Blaney SM, Hancock ML, Kieran MW, Phillips P, et al. Phase I trial of imatinib in children with newly diagnosed brainstem and recurrent malignant gliomas: A Pediatric Brain Tumor Consortium report1. Neuro-Oncol. 2007 Apr 1;9(2):145–60.

4. Ladenstein R, Weixler S, Baykan B, Bleeke M, Kunert R, Katinger D, et al. Ch14.18 antibody produced in CHO cells in relapsed or refractory Stage 4 neuroblastoma patients: A SIOPEN Phase 1 study. mAbs. 2013 Sep;5(5):801–9.

5. Zwaan CM, Rizzari C, Mechinaud F, Lancaster DL, Lehrnbecher T, van der Velden VHJ, et al. Dasatinib in Children and Adolescents With Relapsed or Refractory Leukemia: Results of the CA180-018 Phase I Dose-Escalation Study of the Innovative Therapies for Children With Cancer Consortium. J Clin Oncol. 2013 Jul;31(19):2460–8.

6. Fouladi M, Laningham F, Wu J, O’Shaughnessy MA, Molina K, Broniscer A, et al. Phase I Study of Everolimus in Pediatric Patients With Refractory Solid Tumors. J Clin Oncol. 2007 Oct 20;25(30):4806–12.

7. Champagne MA, Capdeville R, Krailo M, Qu W, Peng B, Rosamilla M, et al. Imatinib mesylate (STI571) for treatment of children with Philadelphia chromosome-positive leukemia: results from a Children’s Oncology Group phase 1 study. Blood. 2004 Nov 1;104(9):2655–60.
