# Supplementary material for: Clinical development success rates and social value of pediatric Phase 1 trials in oncology
Source: PLoS One. 2020 Jun 24;15(6):e0234911. doi: 10.1371/journal.pone.0234911 (PMC7313751; doi:10.1371/journal.pone.0234911)
Supplement: S2 Table — (DOCX) [file pone.0234911.s002.docx]

**S2 Table. Drugs and drug combinations used in included studies, approval status**

| **Author(s) and year(s)** | **Drugs in Phase I trials** | **Drugs approved by FDA before Phase 1 publication** | **Drugs approved by EMA before Phase 1 publication** | **Drugs approved by FDA for pediatric population within 5‑years since Phase 1 publication** | **Drugs approved by EMA for pediatric population within 5‑years since Phase 1 publication** |
| --- | --- | --- | --- | --- | --- |
| **Chemotherapy studies** | | | | | |
| Fox 2006, Fox 2008 | ABT-751 (2 studies) | ✘ | ✘ | ✘ | ✘ |
| Nelken 2012 | Asparaginase, Clofarabine, Dexamethasone, Etoposide, Mitoxantrone | ✓ | ✘ | ✘ | ✘ |
| Malogolowkin 2013 | Bleomycin, Cisplatin, Cyclophosphamide, Etoposide | ✓ | ✘ | ✘ | ✘ |
| Spunt 2007 | Carbamazepine, Oxaliplatin | ✓ | ✘ | ✘ | ✘ |
| Radhakrishnan 2012 | Carboplatin, Ifosfamide, Topotecan | ✓ | ✘ | ✘ | ✘ |
| Levy 2008 | Carboplatin, Irinotecan | ✓ | ✘ | ✘ | ✘ |
| Chintagumpala 2004 | Carboplatin, Thalidomide | ✓ | ✘ | ✘ | ✘ |
| Jakacki 2011 | Carboplatin, Vinblastine* | ✓ | ✘ | N/A | ✘ |
| Adams 2008 | Carmustine (BCNU), O6-Benzylguanine (O6-BG) | ✘ | ✘ | ✘ | ✘ |
| Geoerger 2005 | Cisplatin, Temozolomide | ✓ | ✘ | ✘ | ✘ |
| Inaba 2009 | Cladribine, Topotecan | ✓ | ✓ | ✘ | ✘ |
| Jeha 2004 | Clofarabine | ✘ | ✘ | ✓ | ✓ |
| Elmoneim 2011 | Clofarabine, Cyclophosphamide | ✓ | ✘ | ✘ | ✘ |
| Hijiya 2009 | Clofarabine, Cyclophosphamide, Etoposide | ✓ | ✘ | ✘ | ✘ |
| Cooper 2013 | Clofarabine, Cytarabine | ✓ | ✓ | ✘ | ✘ |
| George 2010 | Cyclophosphamide, Decitabine, Dexrazoxane, Doxorubicin | ✓ | ✘ | ✘ | ✘ |
| Saulnier Sholler 2011 | Cyclophosphamide, Nifurtiomox, Topotecan | ✘ | ✘ | ✘ | ✘ |
| Aquino 2004 | Cyclophosphamide, Tirapazamine | ✘ | ✘ | ✘ | ✘ |
| Casanova 2004 | Cyclophosphamide, Vinorelbine | ✓ | ✘ | ✘ | ✘ |
| Bomgaars 2004 | Cytarabine | ✓ | ✓ | ✘ | ✘ |
| Lowis 2006 | Daunorubicin* | ✓ | ✘ | N/A | ✘ |
| Sholler 2013 | Difluoromethylornithine (DFMO), Etoposide | ✘ | ✘ | ✘ | ✘ |
| Mascarenhas 2013 | Doxorubicin, Oxaliplatin | ✓ | ✘ | ✘ | ✘ |
| Lau 2005 | Ecteinascidin-743 | ✘ | ✘ | ✘ | ✘ |
| McGregor 2008 | Etoposide, Oxaliplatin | ✓ | ✘ | ✘ | ✘ |
| Ruggiero 2010, Ruggiero 2013 | Etoposide, Temozolomide (2 studies) | ✓ | ✘ | ✘ | ✘ |
| Macy 2012 | Fluorouracil, Leucovorin, Oxaliplatin | ✓ | ✘ | ✘ | ✘ |
| Reid 2004 | Gemcitabine | ✓ | ✘ | ✘ | ✘ |
| Geller 2009 | Ifosfamide, Paclitaxel | ✓ | ✘ | ✘ | ✘ |
| Kawamoto 2010 | Ifosfamide, Topotecan | ✓ | ✘ | ✘ | ✘ |
| Bomgaars 2005, Furman 2006, McGregor 2011 | Irinotecan (3 studies) | ✓ | ✘ | ✘ | ✘ |
| Mc Gregor 2009 | Irinotecan, Oxaliplatin | ✓ | ✘ | ✘ | ✘ |
| Wagner 2004, Wagner 2009 | Irinotecan, Temozolomide (2 studies) | ✓ | ✘ | ✘ | ✘ |
| Mc Nall-Knapp 2010, Wagner 2010 | Irinotecan, Temozolomide, Vincristine (2 studies) | ✓ | ✘ | ✘ | ✘ |
| Carlos Rodriguez-Galindo 2005 | Irinotecan, Topotecan | ✓ | ✘ | ✘ | ✘ |
| Bomgaars 2005 | Irofulven | ✘ | ✘ | ✘ | ✘ |
| Widemann 2009 | Ixabepilone | ✓ | ✘ | ✘ | ✘ |
| Blaney 2005 | Mafosfamide | ✘ | ✘ | ✘ | ✘ |
| Kurtzberg 2005 | Nelarabine | ✘ | ✘ | ✓ | ✓ |
| Warren 2005, Broniscer 2007 | O6-Benzylguanine, Temozolomide (2 studies) | ✘ | ✘ | ✘ | ✘ |
| Geoerger 2008 | Oxaliplatin | ✓ | ✘ | ✘ | ✘ |
| Horton 2007 | Paclitaxel | ✓ | ✓ | ✘ | ✘ |
| Malempati 2007 | Pemetrexed | ✓ | ✓ | ✘ | ✘ |
| Georger 2011 | Plitidepsin | ✘ | ✘ | ✘ | ✘ |
| Horton 2005 | Raltitrexed | ✘ | ✘ | ✘ | ✘ |
| Garurangan 2006 | Spartaject Busulfan | ✓ | ✓ | ✘ | ✘ |
| Baruchel 2006, Horton 2007 | Temozolomide (2 studies) | ✓ | ✓ | ✘ | ✘ |
| Rubie 2010 | Temozolomide, Topotecan | ✓ | ✓ | ✘ | ✘ |
| Sauliner Sholler 2011 | Temozolomide, TPI 287 | ✘ | ✘ | ✘ | ✘ |
| Daw 2004, Wagner 2004, Blaney 2012 | Topotecan (3 studies) | ✓ | ✓ | ✘ | ✘ |
| Chuk 2012 | Trabectedin | ✘ | ✓ | ✘ | ✘ |
| Shah 2012 | Vincristine Sulfate Liposomes | ✓ | ✘ | ✘ | ✘ |
| Johansen 2006 | Vinorelbine | ✓ | ✘ | ✘ | ✘ |
| Gururang 2008 | VNP40101M Cloretazine | ✘ | ✘ | ✘ | ✘ |
| **Targeted therapy studies** | | | | | |
| Bagatell 2007, Weigel 2007 | 17-Allylaminogeldanamycin (17-AAG) (2 studies) | ✘ | ✘ | ✘ | ✘ |
| Bender 2012 | Aflibercept | ✓ | ✘ | ✘ | ✘ |
| Mosse 2012 | Alisertib (MLN 8237) | ✘ | ✘ | ✘ | ✘ |
| Fox 2008 | Arsenic trioxide (Trisenox)** | ✓ | ✓ | unclear | ✘ |
| Glade Bender 2008 | Bevacizumab | ✓ | ✓ | ✘ | ✘ |
| Wayne 2010 | BL22 (CAT-3888) | ✘ | ✘ | ✘ | ✘ |
| Blaney 2004, Horton 2007 | Bortezomib (2 studies) | ✓ | ✓/✘*** | ✘ | ✘ |
| Muscal 2012 | Bortezomib, Vorinostat | ✓ | ✘ | ✘ | ✘ |
| Locatelli 2013 | Brentuximab Vedotin | ✓ | ✓ | ✘ | ✘ |
| Fox 2010 | Cediranib | ✘ | ✘ | ✘ | ✘ |
| Ladenstein 2013 | Ch14.18/CHO | ✘ | ✘ | ✓ | ✓ |
| MacDonald 2008 | Cilengitide | ✘ | ✘ | ✘ | ✘ |
| Malempati 2012 | Cixutumumab | ✘ | ✘ | ✘ | ✘ |
| Herrera 2009 | Combotox (HD37 and RFB4 1:1) | ✘ | ✘ | ✘ | ✘ |
| Mosse 2013 | Crizotinib | ✓ | ✓ | ✘ | ✘ |
| Frappaz 2013 | Dalotuzumab | ✘ | ✘ | ✘ | ✘ |
| Zwaan 2013 | Dasatinib | ✓ | ✓ | ✓ | ✓ |
| Fouladi 2006 | Depsipeptide | ✘ | ✘ | ✘ | ✘ |
| Geoerger 2010 | Erlotinib | ✓ | ✓ | ✘ | ✘ |
| Fouladi 2007 | Everolimus | ✘ | ✘ | ✓ | ✘ |
| Villablanca 2006, Maurer 2013 | Fenretinide (2 studies) | ✘ | ✘ | ✘ | ✘ |
| Juergens 2011 | Figitumumab | ✘ | ✘ | ✘ | ✘ |
| Whitlock 2005 | Flavopiridol | ✘ | ✘ | ✘ | ✘ |
| Arceci 2005 | Gemtuzumab ozogamicin** | ✓ | ✘ | unclear | ✘ |
| Osenga 2006 | Hu14.18-IL2 (EMD273063) | ✘ | ✘ | ✘ | ✘ |
| Champagne 2004, Pollack 2007 | Imatinib (2 studies) | ✓ | ✓ | ✓ | ✘ |
| Merchant 2012 | Ipilimumab | ✓ | ✓ | ✘ | ✘ |
| Souid 2010 | Ispinesib | ✘ | ✘ | ✘ | ✘ |
| Fouladi 2010 | Lapatinib | ✓ | ✓ | ✘ | ✘ |
| Geoerger 2012 | LDE225 (Sonidegib) | ✘ | ✘ | ✘ | ✘ |
| Adamson 2004 | Leflunomide (SU101) | ✓ | ✘ | ✘ | ✘ |
| Warren 2011, Berg 2011 | Lenalidomide (2 studies) | ✓ | ✓ | ✘ | ✘ |
| Minturn 2011 | Lestaurtinib | ✘ | ✘ | ✘ | ✘ |
| Merchant 2012 | Lexatumumab | ✘ | ✘ | ✘ | ✘ |
| Kieran 2007 | Lonafarnib | ✘ | ✘ | ✘ | ✘ |
| Fouladi 2011 | MK0752 | ✘ | ✘ | ✘ | ✘ |
| Glade Bender 2013 | Pazopanib | ✓ | ✓ | ✘ | ✘ |
| Becher 2010 | Perifosine | ✘ | ✘ | ✘ | ✘ |
| Zorzi 2013 | Pracinostat (SB939) | ✘ | ✘ | ✘ | ✘ |
| Bagatell 2010 | RG1507 | ✘ | ✘ | ✘ | ✘ |
| Pearson 2013, Gore 2013 | Ridaforolimus (2 studies) | ✘ | ✘ | ✘ | ✘ |
| Widemann 2012 | Sorafenib | ✓ | ✓ | ✘ | ✘ |
| Kieran 2008 | SU5416 (Semaxanib) | ✘ | ✘ | ✘ | ✘ |
| DuBois 2011 | Sunitinib | ✓ | ✓ | ✘ | ✘ |
| Spunt 2011 | Temsirolimus | ✓ | ✓ | ✘ | ✘ |
| Coulter 2013 | Temsirolimus, Valproic Acid | ✓ | ✘ | ✘ | ✘ |
| Widemann 2010 | Tipifarnib | ✘ | ✘ | ✘ | ✘ |
| Leary 2013 | Trebananib (AMG386) | ✘ | ✘ | ✘ | ✘ |
| Su 2010 | Valproic Acid | ✓ | ✘ | ✘ | ✘ |
| Fox 2013 | Vandetanib | ✓ | ✓ | ✘ | ✘ |
| Gajjar 2013 | Vismodegib | ✓ | ✓ | ✘ | ✘ |
| Fouladi 2010 | Vorinostat ± Retinoic Acid | ✓ | ✘ | ✘ | ✘ |
| **Combination therapy studies** | | | | | |
| Santana 2011, Navid 2012 | Bevacizumab, Cyclophosphamide, Sorafenib (2 studies) | ✓ | ✘ | ✘ | ✘ |
| Okada 2013 | Bevacizumab, Irinotecan | ✓ | ✘ | ✘ | ✘ |
| Stapleton 2012 | Bevacizumab, Irinotecan, Temozolomide | ✓ | ✘ | ✘ | ✘ |
| Venkatramani 2013 | Bevacizumab, Irinotecan, Temozolomide, Vincristine | ✓ | ✘ | ✘ | ✘ |
| Messinger 2010 | Bortezomib, Dexamethasone, Doxorubicin, PEG-Asparaginase, Vincristine | ✓ | ✘ | ✘ | ✘ |
| Trippett 2009 | Cetuximab, Irinotecan | ✓ | ✘ | ✘ | ✘ |
| Inaba 2011 | Clofarabine, Cytarabine, Sorafenib | ✓ | ✓ | ✘ | ✘ |
| Rheingold 2007 | Cyclophosphamide, Doxorubicin, G3139 | ✘ | ✘ | ✘ | ✘ |
| Aplenc 2008 | Cytarabine + L-asparaginase, Cytarabine + Mitoxantrone, Gemtuzumab ozogamicin | ✓ | ✘ | ✘ | ✘ |
| Jakacki 2008 | Erlotinib, Temozolomide | ✓ | ✓ | ✘ | ✘ |
| O'Biren 2010 | Etoposide, Mitoxantrone, Valspodar | ✘ | ✘ | ✘ | ✘ |
| Furman 2009 | Gefitinib, Irinotecan | ✓ | ✘ | ✘ | ✘ |
| Bagatell 2013 | Irinotecan, Temozolomide, Temsirolimus | ✓ | ✘ | ✘ | ✘ |
| Marzouki 2012, Morgenstern 2013 | Sirolimus, Vinblastine* (2 studies) | ✓ | ✘ | ✘ | ✘ |
| Hummel 2013 | Temozolomide, Vorinostat | ✓ | ✘ | ✘ | ✘ |

N/A – not applicable; FDA – Food and Drug Administration; EMA – European Medicines Agency

* No label available on FDA website

** the year of pediatrics approval not provided by FDA

*** Bortezomib approved by EMA in 2004
