# Supplementary material for: Clinical development success rates and social value of pediatric Phase 1 trials in oncology
Source: PLoS One. 2020 Jun 24;15(6):e0234911. doi: 10.1371/journal.pone.0234911 (PMC7313751; doi:10.1371/journal.pone.0234911)
Supplement: S3 Table — (DOCX) [file pone.0234911.s003.docx]

Transition, i.e. advancing to further phases of clinical trials, was assessed through systematic search of ClinicalTrials.gov and the EU Clinical Trials databases. This was done in several stages:

1. for every study included in our systematic review we searched both databases for Phase 2, 3 or 4 trial that tested exactly the same drugs or drug combinations in at least one oncological pediatric indication tested in the Phase 1 trial
2. we determined what was the latest phase to have started within five years since the original Phase 1 publication
3. we supplemented the above database search with results from our Google Scholar search for citations – here we identified Phase 2, 3 or 4 pediatric trials that cited Phase 1 trials from our group and tested the same drug/s.

The Phase 1 pediatric trial was classified as transitioned to further phases of testing if we could find at least one relevant Phase 2, 3 or 4 trial in ClinicalTrials.gov, EU Clinical Trials Register or through Google Scholar.

**S3 Table. Transitions status of trials included in the review.**

| **Author and year** | **Drug(s) tested in the study** | **Types of tumors in the study** | **Highest Development Phases of the drug or drugs combination in children within 5 years since publication (tumor types in the study) found in ClinicalTrials.gov** | **Highest Development Phases of the drug or drugs combination in children within 5 years since publication found in EU Clinical Trials Register** | **Number of citations in Google Scholar within 5 years** | | **Transition summary  yes if advanced to further studies** |
| --- | --- | --- | --- | --- | --- | --- | --- |
|  |  |  |  |  | **Pediatric: phase II (same combination)** | **Pediatric: phase III (same combination)** |  |
| Fox 2006 | ABT-751 | Neuroblastoma,  Osteosarcoma, Synoviall cell sarcoma, Erwing’s family tumors, Other sarcoma,Wilms tumor, primary CNS tumour, other | Phase 2; NCT00436852 (Disseminated Neuroblastoma, Recurrent Neuroblastoma) | N/A | 0 | 0 | yes |
| Fox 2008 | ABT-751 | Neuroblastoma, Osteosarcoma, Synoviall cell sarcoma, Erwing’s family tumors, Other sarcoma, other (Papillary thyroid carcinoma) | Phase 2; NCT00436852 (Disseminated Neuroblastoma, Recurrent Neuroblastoma) | N/A | 0 | 0 | yes |
| Nelken 2012 | Asparaginase, Clofarabine, Dexamethasone, Etoposide, Mitoxantrone | Acute Lymphoblastic Leukemia | N/A | N/A | study not cited | study not cited | no |
| Malogolowkin 2013 | Bleomycin, Cisplatin, Cyclophosphamide, Etoposide (C-PEB regimen) | High risk malignant germ cell tumors with either yolk sac tumor (endodermalsinus tumor), embryonal carcinoma, choriocarcinoma, or teratomawith mixed malignant elements | N/A, unclear | N/A | 0 | 0 | no |
| Spunt 2007 | Carbamazepine, Oxaliplatin | Neuroblastoma,Ganglionneuroblasstoma, Medulloblastoma, Hepatocellular carcinoma, colorectar carcinoma, , Erwing’s family tumors, Rhabdomyosarcoma, Wilms tumor,Chondrosarcoma, Ependymoma (anaplastic), Hepatoblastoma, Desmoplastic small round cell tumor, Epithelioid sarcoma, Gliomatosis cerebri | N/A | N/A | 0 | 0 | no |
| Radhakrishnan 2012 | Carboplatin, Ifosfamide, Topotecan | CNS tumors, sarcoma, Wilms tumor, Burkitt lymphoma,hepatoblastoma, neuroblastoma | N/A | N/A | 0 | 0 | no |
| Levy 2009 | Carboplatin, Irinotecan | Astrocytoma 3, Hepatoblastoma 4, Medulloblastoma 2, Neuroblastoma 4, Osteosarcoma 4, Rhabdomyosarcoma 3, Wilms tumor 2, Other, | N/A, further phases started before P1 study | N/A | 1 | 0 | yes |
| Chintagumpala 2004 | Carboplatin, Thalidomide | Malignant glioma (including brainstem glioma) 7, Medulloblastoma 4, Anaplastic ependymoma 2, Ewing’s sarcoma 2, Osteosarcoma 2, Neuroblastoma 2, Wilms’ tumor 1, Renal cell carcinoma 1, Angiosarcoma 1, | N/A, further phases started before P1 study | N/A | 0 | 0 | no |
| Jakacki 2011 | Carboplatin, Vinblastine | juvenile pilocytic astrocytomas, Grade 2 astrocytoma, Low-grade glioma“not otherwise specified | N/A | N/A | 0 | 0 | no |
| Adams 2008 | Carmustine (BCNU), O6-Benzylguanine (O6-BG) | Medulloblastoma,Ependymoma, GBM, Gliomatosis Cerebri; Brain Stem Glioma, Astrocystoma (Anaplastic, Pilocystic, Xanthoastocytoma) | N/A | N/A | 0 | 0 | no |
| Geoerger 2005 | Cisplatin,Temozolomide | CNS tumors (Malignant glioma, Anaplastic ganglioglioma; anapl.plemorphic xanto-astrocytoma; Medulloblastoma, Primitive neuroectodermal tumor; Ependymoma, GBM, Oligodendroglioma, Brain Stem Glioma, ), Non-CNS tumors (Neuroblastoma, Neurofibrosarcoma, Other sarcoma, Rhabdomyosarcoma, Erwing’s tumours, desmoplastic small round cell sarcoma,Melanoma, retinoblastoma,Nephroblastoma) | N/A, further phases started before P1 study | N/A | 0 | 0 | no |
| Inaba 2010 | Cladribine, Topotecan | AML | N/A | N/A | 0 | 0 | no |
| Jeha 2004 | Clofarabine | ALL. AML | Phase 2; NCT00529360 started June 2007 (Acute Lymphoblastic Leukemia, Acute Myelogenous Leukemia) | Phase 2 | 1 | 0 | yes |
| Elmoneim 2012 | Clofarabine, Cyclophosphamide | ALL ,AML | N/A | N/A | 0 | 0 | no |
| Hijiya 2009 | Clofarabine, Cyclophosphamide, Etoposide | ALL i AML | N/A, further phases started before P1 study | N/A | 1 | 0 | yes |
| Cooper 2013 | Clofarabine, Cytarabine | ALL | N/A | N/A | 0 | 0 | no |
| George 2010 | Cyclophosphamide, Decitabine, Dexrazoxane, Doxorubicin | Rhabdomyosarcoma; Osteosarcoma; Neuroblastoma; Synovial sarcoma | N/A | N/A | 0 | 0 | no |
| Saulnier Sholler 2011b | Cyclophosphamide, Nifurtiomox, Topotecan | Neuroblastoma | N/A, further phases started before P1 study | N/A | 0 | 0 | no |
| Aquino 2004 | Cyclophosphamide, Tirapazamine | Neuroblastoma; CNS tumors; Rhabdomyosarcoma; Soft-tissue sarcoma; Hepatoblastoma; Osteosarcoma; Adrenocortical carcinoma; Ewing sarcoma; PNET; Wilms tumor | N/A | N/A | 0 | 0 | no |
| Casanova 2004 | Cyclophosphamide, Vinorelbine | Rhabdomyosarcoma, Malignant peripheral nerve sheath Tumor, Desmoplastic small round cell tumor, SS-synovial sarcoma | N/A | N/A | 0 | 0 | no |
| Bomgaars 2004 | Cytarabine (DepoCyt) | ALL, (NTNB, B-cell) AML,CNS tumors (Medulloblastoma,Unidifferentiated glial tumor) | N/A, unclear | Phase 3 | 0 | 0 | yes |
| Lowis 2006 | Daunorubicin (DaunoXome) | Osteosarcoma, Rhabdomyosarcoma, PNET, Glioma, Ewings, Astrocytoma, Ependymoma, NHL, Neurosarcoma, Hodgkins, Undiff nasopharngeal ca, Hepatocellular carcinoma, Malignant germinal tumour, Malignant adrenal cortical carcinoma, Choroid plexus carcinoma, Neuroblastoma, Synovial sarcoma, Medulloblastoma, Spinal PNET, PPNET, Anaplastic Ependymoma, Clear cell sarcoma, Nephroblastoma | N/A | N/A | 0 | 0 | no |
| Sholler 2013 | Difluoromethylornithine (DFMO), Etoposide | relapsed Neuroblastoma | N/A | N/A | 0 | 0 | no |
| Mascarenhas 2013 | Doxorubicin, Oxaliplatin | Osteosarcoma; Neuroblastoma; Rhabdomyosarcoma; Germ cell tumor; Nasopharyngeal carcinoma; Dentritic cell sarcoma; Wilms tumor; Neurofibrosarcoma; Thymic carcinoma; Ewing sarcoma; Hepatocellular carcinoma | N/A | N/A | 0 | 0 | no |
| Lau 2005 | Ecteinascidin-743 (ET743, trabectedin, Yondelis) | Osteosarcoma 4, Ewing sarcoma 3, Wilms tumor 2, Hepatoblastoma 1, Rhabdomyosarcoma 1, Synovial sarcoma, | Phase 2; NCT00070109 started January 2008 (Previously Treated Childhood Rhabdomyosarcoma Recurrent Childhood Rhabdomyosarcoma Recurrent Childhood Soft Tissue Sarcoma Recurrent Ewing Sarcoma Peripheral Primitive Neuroectodermal Tumor) | N/A | 0 | 0 | yes |
| Mc Gregor 2009 | Oxaliplatin, Irinotecan | Osteosarcoma, Astrocytoma, Renal cell carcinoma, Rhabdomyosarcoma, Ewing sarcoma, Ganglioglioma, Medulloblastoma, Neuroblastoma | N/A | N/A | 0 | 0 | no |
| McGregor 2009 | Etoposide, Oxaliplatin | Ependymoma,Neuroblastoma,Medulloblastoma/supratentorial PNET ,Atypical teratoid rhabdoid tumor,Osteosarcoma,Pineoblastoma, Malignant peripheral nerve sheath tumor,Wilms tumor | N/A | N/A | 0 | 0 | no |
| Ruggiero 2010 | Etoposide, Temozolomide | Medulloblastoma | N/A | N/A | 0 | 0 | no |
| Ruggiero 2013 | Etoposide, Temozolomide | Astroblastoma, Anaplastic Astrocystoma, Glioblastoma, Brainstem glioma | N/A | N/A | study not cited | study not cited | no |
| Macy 2013 | Fluorouracil, Leucovorin, Oxaliplatin | ependymoma, medulloblastoma, chondroma, diffuse pontine glioma, high grade glioma, AT/RT, Hepatoblastoma, Sarcoma, Carcinoid, Lymphoepithelioma, Adenocarcinoma of liver, hepatocellular carcinoma, neuroblastoma | N/A | N/A | 0 | 0 | no |
| Reid 2004 | Gemcitabine | Osteosarcoma 13 31.0 Soft-tissue sarcoma 9 21.4 Ewing’s sarcoma 3 7.1 Rhabdomysosarcoma 3 7.1 Extraosseous Ewing’s sarcoma 2 4.8 Wilms’ tumor 2 4.8 Pancreatic adenocarcinoma 2 | N/A, further phases started before P1 study | N/A | 1 | 0 | yes |
| Geller 2009 | Ifosfamide, Paclitaxel | Alveolar soft part sarcoma; Ewing sarcoma;Neurofibrosarcoma; Hepatoblastoma; Anaplastic ependymoma, Adrenocortical carcinoma Mesothelioma; Neuroblastoma; Osteosarcoma; Synovial cell sarcoma; Wilms’ tumor | N/A | N/A | 0 | 0 | no |
| Kawamoto 2010 | Ifosfamide, Topotecan | Rhabdomyosarcoma, Hepatoblastoma (+ NR?) | N/A | N/A | study not cited | study not cited | no |
| Furman 2006 | Irinotecan | Neuroblastoma, Rhabdomyosarcoma, Brain Tumor, Erwing sarcoma family of tumors, cell carcinomas, hemangiopericytoma, germ cell tumor, hepatoblastoma, alveolar soft-part sarcoma, undifferentiated sarcoma, osteosarcoma, malignant schwannoma, chordoma | N/A, further phases started before P1 study | N/A | 1 | 0 | yes |
| McGregor 2012 | Irinotecan (+ adjuvant oral Cefpodoxime) | Neuroblastoma, Wilms Tumor, Ewing Sarcoma, Nasopharyngeal carcinoma, Malignant Peripheral Nerrve Sheath Tumor, Paraganglioma, Renal cell carcinoma, Chondroma, High grade sarcoma | N/A, further phases started before P1 study | N/A | 0 | 0 | no |
| Bomgaars 2006a | Irinotecan | Non-CNS tumors (Hepatoblastoma, Synovial sarcoma soft tissue sarcomas, Hepatic sarcoma, Neuroblastoma, Rhabdomyosarcoma), CNS tumors (Ependymoma, Optic glioma, Pontine glioma) | N/A, further phases started before P1 study | Phase 2 | 3 | 0 | yes |
| Wagner 2004 | Irinotecan, Temozolomide | Ewing’s sarcoma, Neuroblastoma, Wilms tumor, Ependymoma,Low-grade glioma | Phase 2; NCT00311584 started April 2006, NCT00404495 started April 2007 (Neuroblastoma, Glioma, Medulloblastoma, Brain and Central Nervous System Tumors) | Phase 2 | 0 | 0 | yes |
| Wagner 2009 | Irinotecan, Temozolomide | High risk neuroblastoma | Phase 2; NCT00311584 started April 2006, NCT00404495 started April 2007 (Neuroblastoma, Glioma, Medulloblastoma, Brain and Central Nervous System Tumors) | N/A | 2 | 0 | yes |
| Mc Nall-Knapp 2010 | Irinotecan, Temozolomide, Vincristine | Neuroblastoma (Bone marrow involvement) Osteosarcoma,Brainstem glioma,Hepatoblastoma,Ewing sarcoma,Ependymoma,Nasopharyngeal carcinoma,Undifferentiated sarcoma,Desmoplastic small round cell tumor,Hepatocellular carcinoma,Optic pathway glioma,Rhabdomyosarcoma,Rhabdoid tumor of kidney | Phase 2; NCT01355445 started January 2012 (RHABDOMYOSARCOMA) | N/A | 0 | 0 | yes |
| Carlos Rodriguez-Galindo 2006 | Irinotecan,Topotecan | Neurooblastoma, Gliomatosis, Nasopharyngeal carcinoma, Osteosarcoma, Rhabdomyosarcoma | N/A | N/A | 0 | 0 | no |
| Bomgaars 2006b | Irofulven (MGI 114) | Adenocarcinoma colon 2, Glioblastoma 1, Hepatoblastoma 2, Hepatocellular carcinoma 1, Juvenile granulosa cell tumor 1, Neuroblastoma 3, Osteosarcoma 9, PNET 1, Poorly differentiated eyelid carcinoma 1, Primitive neuroepithelial brain tumor 1, Renal cell carcinoma 1, Rhabdomyosarcoma 7, Spindle cell sarcoma 1, Synovial cell sarcoma 1, Undifferentiated embryonal sarcoma 1, Wilms tumor, | N/A | N/A | 0 | 0 | no |
| Widemann 2009 | Ixabepilone (BMS-247550, NSC 710428) | Rhabdomyosarcoma 3, Other soft tissue sarcoma (NR) 4, Osteosarcoma 3, Ewing sarcoma 2, Hepatoblastoma 2, Wilms’ tumor 2, Neuroblastoma 1, Other (NR), | Phase 4; NCT00866671 started February 2009 (Leukaemia, Lymphoblastic, Acute) | N/A | 1 | 0 | yes |
| Blaney 2005 | Mafosfamide | Medulloblastoma, ATRT, PNET, Ependymoma, Pineoblastoma, Choroid plexus papilloma | N/A | N/A | 0 | 0 | no |
| Kurtzberg 2005 | Nelarabine (506U78) | T-ALL/LBL;T-NHL;T-CLL/T-PLL/T-LPD;B-ALL/pre-B-ALL;B-NHL;AML/CML-BC;Other unknown immunophenotype or biphenotypic leukemia | Phase 4; NCT00866671 started February 2009 (Leukaemia, Lymphoblastic, Acute) | N/A | 0 | 0 | yes |
| Warren 2005 | O6-Benzylguanine, Temozolomide | Sarcoma,CNS tumors:High-grade glioma,Low-grade glioma,Brainstem glioma, Medulloblastoma/PNET,Ependymoma | Phase 2; NCT00275002 started February 2006 (Brain and Central Nervous System Tumors) | N/A | 0 | 0 | yes |
| Broniscer 2007 | O6-Benzylguanine, Temozolomide | Malignant glioma , Brain stem glioma , Medulloblastoma , PNET , Ependymoma , Atypical teratoid rhabdoid tumor , Low-grade glioma, | N/A, further phases started before P1 study | N/A | 1 | 0 | yes |
| Wagner 2010 | Oral Irinotecan, Temozolomide, Vincristine | Ewing sarcoma 5;Osteosarcoma 3;Rhabdomyosarcoma 6;Synovial sarcoma 2;Spindle cell sarcoma 1;Undifferentiated sarcoma 1;Alveolar soft parts sarcoma 1;Neuroblastoma 2;Hepatolastoma 3;Wilms tumor 2;Ependymoma 6;Medulloblastoma 2;Malignant glioma 4;Fibrillary astrocytoma 1;Atypical teratoid rhabdoid tumor 1;Paraganglioma 1;Pleuropulmonary blastoma 1 | Phase 2; NCT01355445 started January 2012 (RHABDOMYOSARCOMA) | N/A | 0 | 0 | yes |
| Geoerger 2008 | Oxaliplatin | Neuroblastoma Osteosarcoma Ewing’s tumor Nephroblastoma Rhabdomyosarcoma Germ cell tumor Brain tumors Hepatoblastoma Other malignant tumor (NR) | Phase 2; NCT01558453 started March 2011, NCT00047177 started October 2002, NCT00091182 started October 2004 (Relapsed Solid Tumor, Refractory Solid Tumor,Brain and Central Nervous System Tumors, Childhood Central Nervous System Germ Cell Tumor Childhood Extragonadal Germ Cell Tumor, Childhood Hepatoblastoma, Childhood Hepatocellular Carcinoma Childhood High-grade Cerebral Astrocytoma, Childhood Low-grade Cerebral Astrocytoma Childhood Malignant Ovarian Germ Cell Tumor Childhood Malignant Testicular Germ Cell Tumor Childhood Teratoma, Recurrent Adrenocortical Carcinoma, Recurrent Childhood Brain Stem Glioma Recurrent Childhood Cerebellar Astrocytoma Recurrent Childhood Cerebral Astrocytoma, Recurrent Childhood Ependymoma Recurrent Childhood Liver Cancer Recurrent Childhood Malignant Germ Cell Tumor Recurrent Childhood Rhabdomyosarcoma, Recurrent Childhood Soft Tissue Sarcoma Recurrent Childhood Visual Pathway and Hypothalamic Glioma Recurrent Colon Cancer, Recurrent Ewing Sarcoma/Peripheral Primitive Neuroectodermal Tumor Recurrent Nasopharyngeal Cancer, Recurrent Neuroblastoma Recurrent Osteosarcoma, Recurrent Rectal Cancer,Recurrent Renal Cell Cancer | N/A | 0 | 0 | yes |
| Horton 2008 | Paclitaxel | ALL; AMLa (Two patients had acute promyelocytic leukemia); Biphenotypic; JMML | N/A, further phases started later than 5 years since P1 study | N/A | 0 | 0 | no |
| Malempati 2007 | Pemetrexed | Osteosarcoma 12, Ewing sarcoma 3, Brainstem tumor 3, Hepatoblastoma 2, Renal cell carcinoma 2, Anaplastic astrocytoma or glioblastoma multiforme 2, Glioma 1, Ependymoma 1, Medulloblastoma 1, Pilocytic astrocytoma 1, Nasopharyngeal adenocarcinoma 1, Rhabdomyosarcoma 1, Wilms’ tumor 1, Liposarcoma 1, GI stromal tumor, | Phase 2; NCT00520936 started September 2007 (Osteosarcoma Medulloblastoma Sarcoma, Ewing's Neuroblastoma (Measurable Disease) Neuroblastoma (Metaiodobenzylguanidine Positive Evaluable) Rhabdomyosarcoma Ependymoma Non-brainstem High-grade Glioma) | N/A | 0 | 0 | yes |
| Georger 2012 | Plitidepsin | Neuroblastoma, Nephroblastoma, Osteosarcoma, Ewing’s sarcoma, Medulloblastoma, Rhabdoid tumour, Rhabdomyosarcoma, Anaplastic astrocytoma, Ependymoma, Hepatoblastoma, Other sarcoma, Pancreatoblastoma, Choriod plexus carcinoma, Glioblastoma, Liponeurocytoma, Brain stem glioma | N/A | N/A | 0 | 0 | no |
| Horton 2005 | Raltitrexed (Tomudex, ZD1694, NSC 639186) | ALL 9 AML 8 JCML | N/A | N/A | 0 | 0 | no |
| Garurangan 2006 | Spartaject Busulfan | PNET 15, Ependymoma 4, Malignant glioma 3, Choroid plexus carcinoma, | N/A | N/A | 0 | 0 | no |
| Baruchel 2006 | Temozolomide | Low-grade gliomas , High-grade gliomas , PNET/medulloblastoma , Brain-stem glioma , Meningioma , Ependymoma, | N/A, further phases started later than 5 years since and before P1 study | Phase 2 | 0 | 0 | yes |
| Horton 2007 | Temozolomide | ALL, AML | Phase 2; terminated NCT01857752 started March 2012, NCT00276679 started April 2003, NCT00005955 started August 2000 (Neuroblastoma, Medulloblastoma, Retinoblastoma, Brain and Central Nervous System Tumors) | Phase 2 | 0 | 0 | yes |
| Rubie 2010 | Temozolomide, Topotecan | Rhabdomyosarcoma; Osteosarcoma; High grade glioma; Granulosa cell tumor; UCNT; Neuroblastoma; Ependymoma;Peritoneal carcinomatosis | N/A, further phases started before P1 study | N/A | 1 | 0 | yes |
| Sauliner Sholler 2011a | Temozolomide, TPI 287 | Neuroblastoma, Medulloblastoma | N/A | N/A | study not cited | study not cited | no |
| Daw 2004 | Topotecan | Brain tumor, Osteosarcoma, Neuroblastoma, , Heptoblastoma, Rhabdomyosarcoma, Hepatocellular carcinoma, Erwing Sarcoma, Wilms tumor, Chondrosarcoma | Phase 2; NCT00005811 started April 2000, NCT00112619 started August 2005; NCT00003745 started May 1999 (Leukemia, Neuroblastoma, Brain Tumors, Solid Tumors, Sarcoma, AIDS-related Diffuse Large Cell Lymphoma AIDS-related Diffuse Mixed Cell Lymphoma AIDS-related Diffuse Small Cleaved Cell Lymphoma AIDS-related Immunoblastic Large Cell Lymphoma AIDS-related Lymphoblastic Lymphoma AIDS-related Peripheral/Systemic Lymphoma AIDS-related Primary CNS Lymphoma AIDS-related Small Noncleaved Cell Lymphoma Childhood Diffuse Large Cell Lymphoma Childhood Immunoblastic Large Cell Lymphoma HIV-associated Hodgkin Lymphoma Leptomeningeal Metastases Recurrent Childhood Acute Lymphoblastic Leukemia Recurrent Childhood Acute Myeloid Leukemia Recurrent Childhood Large Cell Lymphoma Recurrent Childhood Lymphoblastic Lymphoma Recurrent Childhood Medulloblastoma Recurrent Childhood Small Noncleaved Cell Lymphoma Recurrent/Refractory Childhood Hodgkin Lymphoma Unspecified Childhood Solid Tumor, Protocol Specific) | N/A | 0 | 0 | yes |
| Wagner 2004 | Topotecan | Recurrent or Progressive High-Grade Glioma (pons tumorsgliomatosis cerebri, anaplastic astrocytoma, glioblastoma multiforme) | Phase 2; NCT00005811 started April 2000, NCT00112619 started August 2005; NCT00003745 started May 1999 (Leukemia, Neuroblastoma, Brain Tumors, Solid Tumors, Sarcoma, AIDS-related Diffuse Large Cell Lymphoma AIDS-related Diffuse Mixed Cell Lymphoma AIDS-related Diffuse Small Cleaved Cell Lymphoma AIDS-related Immunoblastic Large Cell Lymphoma AIDS-related Lymphoblastic Lymphoma AIDS-related Peripheral/Systemic Lymphoma AIDS-related Primary CNS Lymphoma AIDS-related Small Noncleaved Cell Lymphoma Childhood Diffuse Large Cell Lymphoma Childhood Immunoblastic Large Cell Lymphoma HIV-associated Hodgkin Lymphoma Leptomeningeal Metastases Recurrent Childhood Acute Lymphoblastic Leukemia Recurrent Childhood Acute Myeloid Leukemia Recurrent Childhood Large Cell Lymphoma Recurrent Childhood Lymphoblastic Lymphoma Recurrent Childhood Medulloblastoma Recurrent Childhood Small Noncleaved Cell Lymphoma Recurrent/Refractory Childhood Hodgkin Lymphoma Unspecified Childhood Solid Tumor, Protocol Specific) | N/A | 0 | 0 | yes |
| Blaney 2013 | Topotecan | Acute Leukemia, ATRT,Ependymoma,Germinoma,Glioblastoma,multiforme,Medulloblastoma ,Pilocytic astroctyoma, Pineoblastoma | N/A, further phases started before P1 study | N/A | 0 | 0 | no |
| Chuk 2012 | Trabectedin (Yondelis, ET-743) | Desmoplastic small round cell tumor; Embryonal liver sarkoma; Synovial sarkoma; Neuroendocrine carcinoma; Nasopharyngeal carcinoma; Anorexia, fatigue; Osteosarcoma; Osteosarcoma; Ewing’s sarkoma; Malignant peripheral nerve sheath tumor; Osteosarcoma; Dehydration; Diffuse intrinsic pontine; glioza; Mesenchymal chondrosarcoma | N/A, further phases started before P1 study | N/A | 0 | 0 | no |
| Shah 2012 | Vincristine Sulfate Liposomes (VSLI, Marqibo) | ALL, solid tumors | N/A, further phases started before P1 study | N/A | 0 | 0 | no |
| Johansen 2006 | Vinorelbine (Navelbine) | CNS; Sympathetic nervous system; Soft tissue; Bone; Hematologic (one patient with Ki-1 non-Hodgkin’s lymphoma), Nasopharyngeal carcinoma | N/A, further phases started later than 5 years since and before P1 study | N/A | 1 | 0 | yes |
| Gururang 2008 | VNP40101M Cloretazine | Malignant glioma, Brain Stem Glioma, Medulloblastoma, PNET, Ependymoma, Atypical teratoid rhabdoid tumor, Low grade glioma | N/A | N/A | 0 | 0 | yes |
| Santana 2011 | Bevacizumab, Cyclophosphamide, Sorafenib | Refractory solid tumors | N/A | N/A | 0 | 0 | no |
| Navid 2013 | Bevacizumab, Cyclophosphamide, Sorafenib | Rhabdoid tumor;Neuroblastoma;Osteosarcoma;Rhabdomyosarcoma; Synovial sarcoma;aWilms tumor;malignant peripheral nerve sheath tumor;adrenocortical carcinoma;epithelioid sarcoma epithelioid sarcoma; medulloblastoma | N/A | N/A | 0 | 0 | no |
| Okada 2013 | Bevacizumab, Irinotecan | diffuse intrinsie pontine glioma; gliomatosis cerebri; rhabdomyosarcoma; neuroblastoma; optic nerve glioma; ependymoma | N/A | N/A | 0 | 0 | no |
| Stapleton 2012 | Bevacizumab, Irinotecan, Temozolomide | high-grade glioma, low-grade glioma, PNET, ependymoma, and choroid plexus carcinoma | N/A, further phases started before P1 study | Phase 2 | study not cited | study not cited | yes |
| Venkatramani 2013 | Bevacizumab, Irinotecan, Temozolomide, Vincristine | Wilms tumor, osteosarcoma, hepatocellular carcinoma, Ewing sarcoma, medulloblastoma, liposarcoma, synovial sarcoma, angiosarcoma | N/A | N/A | 0 | 0 | no |
| Messinger 2010 | Bortezomib, Dexamethasone, Doxorubicin, PEG-Asparaginase, Vincristine | B-precursor ALL; T-cell ALL | N/A | N/A | 0 | 0 | no |
| Trippett 2009 | Cetuximab, Irinotecan | Ependymoma; Glioma, brainstem; Glioma, high grade; Ewing sarcoma; Hepatoblastoma; Neuroblastoma; Osteosarcoma; Rhabdomyosarcoma; Wilms tumor; chordoma, high-grade neuroepithelial neoplasm, pineal neuroectodermal tumor, atypical teratoid rhabdoid tumor, anaplastic astrocytoma, and choroid plexus carcinoma; malignant peripheral nerve sheath tumor, desmoplastic small round-cell tumor, retinoblastoma, epithelioid sarcoma, lymphoepithelioma, nasopharyngeal carcinoma, temporal bone mesenchymal chondrosarcoma, and metastatic signet ring adenocarcinoma | Phase 2 NCT01012609 started November 2009 (Brain Cancer) | N/A | 0 | 0 | yes |
| Inaba 2011 | Clofarabine, Cytarabine, Sorafenib | acute myeloid leukemia, early T-cell precursor leukemia | N/A | N/A | 0 | 0 | no |
| Rheingold 2007 | Cyclophosphamide, Doxorubicin, G3139 (bcl-2 antisense oligonucleotide) | Ewing’s sarcoma; Osteosarcoma; Nephroblastoma; Neuroblastoma; Synovial sarcoma; Hepatoblastoma; Juvenile granulosa cell tumor; Rhabdomyosarcoma; Thymoma; Adenocarcinoma; Desmoplastic small round cell tumor; Undifferentiated embryonal sarcoma | N/A | N/A | 0 | 0 | no |
| Aplenc 2008 | Cytarabine + L-asparaginase, Cytarabine + Mitoxantrone, Gemtuzumab ozogamicin (GTMZ) | AML (induction failure; early relapse; as a second malignant neoplasm); Myelodysplastic syndrome | N/A | N/A | 0 | 0 | no |
| Jakacki 2008 | Erlotinib, Temozolomide | Brainstem glioma, Medulloblastoma, Supratentorial PNET (primitive neuroectodermal tumor), Ependymoma, Glioblastoma, Neurocytoma, Gliomatosis cerebri, Rhabdomyosarcoma, Soft tissue sarcoma, Neuroblastoma, Osteosarcoma, Germ cell, Rhabdoid | N/A | N/A | 0 | 0 | no |
| O'Biren 2010 | Etoposide, Mitoxantrone, Valspodar (PSC-833) | DS-AML;AML;T-cell ALL (post-B-precursor ALL); Biphenotypic;B-precursor ALL;tAML (post-JMML) | N/A | N/A | 0 | 0 | no |
| Furman 2009 | Gefitinib, Irinotecan | Osteosarcoma; Wilms’ tumor; Brain tumor; Ewing sarcoma family of tumors; *Hepatoblastoma, hepatocellular carcinoma;, adrenocortical carcinoma, undifferentiated sarcoma, polyphenotypic sarcoma. | N/A | N/A | 1 | 0 | yes |
| Marzouki 2012 | Sirolimus, Vinblastine | recurrent or refractory solid tumor icluding CNS tumor | N/A | N/A | study not cited | study not cited | no |
| Hummel 2013 | Temozolomide, Vorinostat | choroid plexus carcinoma, PNET, high grade glioma, ependymoma, medulloblastoma, ganglioglioma, atypical teratoid/rhabdoid tumor | N/A | N/A | 0 | 0 | no |
| Bagatell 2014 | Irinotecan, Temozolomide, Temsirolimus | Neuroblastoma;Osteosarcoma;Ewing family tumor;Rhabdomyosarcoma; Hepatoblastoma; Hepatocellular carcinoma; Glioma;Ependymoma;Medulloblastoma; Primitive neuroectodermal tumor and other (NR) | N/A | N/A | 2 | 0 | yes |
| Morgenstern 2014 | Sirolimus, Vinblastine | neuroblastoma, RMS , embryonal sarcoma, osteosarcoma, Ewing sarcoma, glioma | N/A | N/A | 0 | 0 | no |
| Bagatell 2007 | 17-Allylaminogeldanamycin (17-AAG) | Neuroblastoma; Osteosarcoma; Ewing’s sarcoma; Desmoplastic small round cell tumor | N/A | N/A | 0 | 0 | yes |
| Weigel 2007 | 17-Allylaminogeldanamycin (17-AAG) | Atypical teratoid/rhabdoid tumor; Ependymoma; Ewing sarcoma; Juvenile granulosa cell tumor; Hepatoblastoma; Wilms’ tumor; Neuroblastoma; Peripheral nerve sheath tumor; Osteosarcoma; Primitive neuroectodermal tumor (CNS); Renal cell carcinoma; Synovial sarcoma | N/A | N/A | 0 | 0 | yes |
| Bender 2012 | Aflibercept | Embryonal (Hepatoblastoma;Neuroblastoma;Wilms tumor), Sarcoma (Ewing sarcoma;Alveolar rhabdomyosarcoma;Synovial sarcoma;Other soft tissue sarcoma), Carcinoma (Adrenocortical, Hepatocellular carcinoma, Small cell:large cell carcinoma), Brain tumor (Ependymoma, Pilocytic astrocytoma) | N/A | N/A | 0 | 0 | no |
| Mosse 2012 | Alisertib (MLN 8237) | Ewings sarcoma; Hepatoblastoma; Osteosarcoma; Wilms tumor; Neuroblastoma; Renal cell carcinoma; Rhabdomyosarcoma; Soft tissue sarcomas | N/A, further phases started before P1 study | N/A | 0 | 0 | no |
| Fox 2008 | Arsenic trioxide (Trisenox) | Acute promyelocytic leukemia; Acute lymphoblastic leukemia; Acute myeloid leukemia; B-cell lyphoma | N/A | N/A | 0 | 0 | no |
| Glade Bender 2008 | Bevacizumab | Alveolar soft part sarcoma,Clear cell sarcoma of kidney,Ewing sarcoma,Fibrosarcoma, Hepatoblastoma,Mesenchymal chondrosarcoma,Neuroblastoma,Osteosarcoma,Rhabdoid tumor, extrarenal,Synovial sarcoma,Wilms’ tumor | N/A | N/A | 0 | 0 | yes |
| Wayne 2010 | BL22 (CAT-3888, FB4(dsFv)-PE38) | ALL; Burkitt Leukemia; Lymphoblastic lymphoma | N/A | N/A | 0 | 0 | no |
| Blaney 2004 | Bortezomib | Optic glioma;Osteosarcoma;Hepatoblastoma; Neuroblastoma; Adenocarcinoma; Wilms’ tumor; Rhabdomyosarcoma | N/A | N/A | 0 | 0 | yes |
| Horton 2007 | Bortezomib | AML; (pre-B) ALL | N/A | N/A | 0 | 0 | yes |
| Muscal 2013 | Bortezomib, Vorinostat | Malignant glioma, Malignant peripheral nerve sheath tumor, Medulloblastoma, Neuroblastoma, Extrarenal rhabdoid tumor, Ewing sarcoma, Rhabdomyosarcoma, Hepatoblastoma, Osteosarcoma, Wilms tumor, Embryonal sarcoma, Carcinoma (undifferentiated), Epitheliod sarcoma, Retinoblastoma | N/A | N/A | 0 | 0 | no |
| Locatelli 2013 | Brentuximab Vedotin | Hodgkin lymphoma, systemic anaplastic large cell lymphoma | N/A | Phase 2 | study not cited | study not cited | yes |
| Fox 2010 | Cediranib | Ewing sarcoma family tumors, osteosarcoma, synovial cell sarcoma, alveolar soft part sarcoma, Wilms tumor, other (NR) | N/A, further phases started before P1 study | N/A | 0 | 0 | yes |
| Ladenstein 2013 | Ch14.18 CHO (APN311, dinutuximab beta) | recurrent/refractory neuroblastoma | N/A | Phase 2 | 0 | 0 | yes |
| MacDonald 2008 | Cilengitide | CNS:Astrocytoma, anaplastic;Astrocytoma, NOS;Brainstem glioma; Choroid plexus papilloma, malignant; Ependymoma, anaplastic;Ependymoma, NOS;Fibrillary astrocytoma;Glioblastoma multiforme;Glioma, malignant;Glioma, NOS (except nasal glioma, not neoplastic);Medulloblastoma, NOS;Meningioma, NOS;Oligodendroglioma, anaplastic;Oligodendroglioma, NOS;Primitive neuroectodermal tumor | N/A | N/A | 0 | 0 | yes |
| Malempati 2012 | Cixutumumab | Ewing sarcoma/peripheral PNET; Osteosarcoma; Rhabdomyosarcoma; Wilms tumor; Alveolar soft part sarcoma; Clear cell sarcoma; Epithelioid sarcoma; Fibrosarcoma; Spindle cell sarcoma | N/A, further phases started before P1 study | N/A | 1 | 0 | yes |
| Herrera 2009 | Combotox (HD37 and RFB4 1:1) | B-lineage Acute lymphoblastic leukemia | N/A | N/A | 0 | 0 | no |
| Mosse 2013 | Crizotinib | solid tumors (Ewing's sarcoma, inflammatory myofibroblastic tumor, osteosarcoma, neuroblastoma, non-small-cell lung tumor, hepatocellular carcinoma, Wilms' tumor, malignant schwannoma, fusiform malignancy, rhabdomyosarcoma, soft-tissue sarcoma), anaplastic large-cell lymphoma, CNS tumors (glioma, ependymoma) | N/A | Phase 2 | 1 | 0 | yes |
| Frappaz 2013 | Dalotuzumab | Advanced solid tumors | N/A | N/A | 0 | 0 | no |
| Zwaan 2013 | Dasatinib | CML; Ph positive ALL, PH negative ALL; Ph negative AML | N/A | N/A | 1 | 0 | yes |
| Fouladi 2006 | Depsipeptide | Alveolar soft part sarcoma, Embryonal rhabdomyosarcoma, Ependymoma, Ewing sarkoma, Hepatoblastoma, Medulloblastoma, Nephroblastoma, Neuroblastoma, Osteosarcoma, Pleomorphic rhabdomyosarcoma, Peripheral primitive neuroectodermal tumor, Rhabdomyosarcoma, Synovial sarkoma, Malignant germ cell tumor (yolk sac), Triton tumor | N/A, further phases started before P1 study | N/A | 0 | 0 | no |
| Geoerger 2011 | Erlotinib | glioblastoma, oligodendroglioma, anaplastic oligodendroglioma, anaplastic astrocytoma, gliomatosis, infiltrative brainstem glioma, anaplastic ependymoma, medulloblastoma, cerebral primitive neuroectodermal tumor, myxopapillary ependymoma, choroid plexus papilloma, choroid plexus carcinoma, exophytic brainstem glioma | N/A | Phase 2 | 1 | 0 | yes |
| Fouladi 2007 | Everolimus | Astrocytoma low grade; Astrocytoma high grade (AA, GBM); Brainstem glioma; Atypical teratoid rhabdoid tumor; Ependymoma; Ganglioglioma; Neuroblastoma; Oligoastrocytoma; Osteosarcoma; Primitive neuroectodermal tumor/medulloblastoma; Rhabdomyosarcoma; Sarcoma clear cell | N/A | Phase 3 | 0 | 0 | yes |
| Villablanca 2006 | Fenretinide | Brain tumor, Osteosarcoma, Neuroblastoma, Medulloblastoma, Heptoblastoma, Rhabdomyosarcoma, Glioma, Erwing Sarcoma, Wilms tumor, Melanoma | N/A | N/A | 0 | 0 | yes |
| Maurer 2013 | Fenretinide | Relapsed/Refractory Neuroblastoma (MIBG disease only; Bone marrow only; Soft tissue only; MIBG + bone marrow; Soft tissue = MIBG 7; Soft tissue = MIBG + bone marrow) | N/A | N/A | 0 | 0 | no |
| Juergens 2011 | Figitumumab | Ewing Sarcoma; Osteosarcoma; other sarcoma (NR) | N/A | N/A | 0 | 0 | yes |
| Whitlock 2005 | Flavopiridol (alvocidib) | Hepatocellular carcinoma; Melanocytoma of eyeball; Malignant mesenchymal tumor; Malignant fibrosarcoma; Rhabdomyosarcoma; Wilms’ tumor; Hepatoblastoma; Osteosarcoma; Ewing’s sarcoma; Astrocytoma; Astrocytoma, anaplastic; Primitive neuroectodermal tumor; Ganglioneuroblastoma; Neuroblastoma; Undifferentiated sarcoma; Desmoplastic small round cell tumor; Spindle cell rhabdomyosarcoma; Atypical teratoid/rhabdoid tumor | N/A, further phases started before P1 study | N/A | 0 | 0 | no |
| Arceci 2005 | Gemtuzumab ozogamicin | CD33+ AML | Phase 3; NCT00476541 started January 2004 (Acute Myeloid Leukemia, Leukemia) Phase 4; NCT01041040 started October 2007 (Acute Myeloblastic Leukemia) | N/A | 1 | 0 | yes |
| Osenga 2006 | Hu14.18-IL2 (EMD273063) | Neuroblastoma; Melanoma | N/A, further phases started before P1 study | N/A | 1 | 0 | yes |
| Pollack 2007 | Imatinib | Astrocytoma, anaplastic; Anaplastic ganglioglioma (only stratum IIA); Glioblastoma multiforme; Glioma, malignant; Gliosarcoma (only stratum IIa); Oligodendroglioma, anaplastic (only stratum IIb); Unconfirmed (based on imaging) (without stratum IIb) | N/A | Phase 4 | 0 | 0 | yes |
| Champagne 2004 | Imatinib Mesylate (STI571) | Philadelphia chromosome-positive leukemia:CML,ALL,AML | N/A | Phase 4 | 3 | 0 | yes |
| Merchant 2012a | Ipilimumab | melanoma; osteosarcoma; soft tissue sarcomas; neuroblastoma; renal cell carcinoma | N/A | Phase 2 | 0 | 0 | yes |
| Souid 2010 | Ispinesib | soft tissue sarcoma, rhabdomyosarcoma, Wilms tumor, hepatoblastoma, lymphoepithelial carcinoma, adenocarcinoma, neuroendocrine hepatic carcinoma, pancreatoblastoma, malignant glioma, astrocytoma, ependymoma, atypical teratoid/rhabdoid tumor | N/A | N/A | 0 | 0 | no |
| Fouladi 2010b | Lapatinib | Astrocytoma (NOS), Anaplastic ganglioglima, Brainstem glioma; Ependymoma, High-grade glioma (glioblastoma, multiforme, anaplastic astrocytoma); Glimastosis cerebri, Medulloblastoma/primitive neuroectodermal tumor; Pineoblastoma; Pleomorphic xanthoastrocytoma, Atypical teradoid rhabdoid tumor) | N/A | N/A | 1 | 0 | yes |
| Geoerger 2012 | LDE225 (Sonidegib) | meduloblastoma; rhabdomyoscarcoma; osteosarcoma; neuroblastoma, gliomatosis; glioblastoma; oligoastrocytoma | N/A | N/A | 0 | 0 | no |
| Adamson 2004 | Leflunomide (SU101) | Ewing’s sarcoma; Medulloblastoma; Ependymoma; Glioma; Osteosarcoma; Brain stem glioma; Desmoplastic small round-cell tumor; Neuroblastoma; Alveolar soft-part sarcoma; Esthesioneuroblastoma; Glioblastoma multiforme; Melanoma of the soft parts | N/A | N/A | 0 | 0 | no |
| Warren 2011 | Lenalidomide | high-grade glioma, brain-stem glioma; low-grade glioma; primitive neuroectodermal tumor/medulloblastoma, ependymoma, other (NR) | N/A | Phase 2 | 0 | 0 | yes |
| Berg 2011 | Lenalidomide | Nonrhabdomyosarcoma soft tissue sarcoma; Osteosarcoma, Ewing's sarcoma; Carcinoma; Rhabdomyosarcoma; Malignant peripheral nerve sheath tumor; Myelodysplastic syndrome; Hepatoblastoma; Nephroblastoma; Alveolar soft part sarcoma; Atypical teratoid/rhabdoid tumor; Hepatocellular carcinoma; Hodgkin's disease; Neuroblastoma; Pleuropulmonary blastoma | N/A | Phase 2 | 0 | 0 | yes |
| Minturn 2011 | Lestaurtinib | neuroblastoma - 47 | N/A | N/A | 0 | 0 | no |
| Merchant 2012b | Lexatumumab | Ewing sarcoma; Osteosarcoma; Rhabdomyosarcoma; Synovial sarcoma; Alveolar soft part sarcoma; Undifferentiated sarcoma; Hepatoblastoma; Nephroblastoma; pindle epithelial tumor with thymus-like differentiation | N/A | N/A | 0 | 0 | no |
| Kieran 2007 | Lonafarnib | Astrocytoma, anaplastic; Astrocytoma, NOS; Anaplastic ganglioglioma; Brain stem glioma; Cerebellar sarcoma NOS; Desmoplastic medulloblastoma; Ependymoma, anaplastic; Ependymoma NOS; Ganglioglioma; Gangliocytoma;Glioblastoma multiforme; Glioma, malignant; Gliomatosis cerebri; Juvenile astrocytoma; Medulloblastoma NOS; Meningioma, malignant; Meningioma NOS; Mixed glioma; Myxopapillary ependymoma; Primitive neuroectodermal tumor, Oligodendroglioma | N/A | N/A | 0 | 0 | no |
| Fouladi 2011 | MK0752 | Ependymoma, Brain stem glioma, Medulloblastoma/primitive neuroectodermal tumor Glioblastoma multiforme, Malignant glioma (grade III to IV), Choroid plexus carcinoma Atypical teratoid/rhabdoid tumor | N/A | N/A | 0 | 0 | no |
| Glade Bender 2013 | Pazopanib | sarcomas (rhabdomyosarcoma, osteosarcoma, synovial sarcoma, Ewing sarcoma, alveolar soft part sarcoma, clear cell sarcoma, desmoplastic small round cell, other soft tissue sarcoma); brain tumor (high-grade glioma, ependymoma, low-grade glioma, germ cell, medulloblastoma/PNET, atypical teratoid/rhabdoid), embryonal (hepatoblastoma, Wilms tumor), other (melanoma, renal cell) | N/A | Phase 2 | 0 | 0 | yes |
| Becher 2010 | Perifosine | grade glioma (n=5), medulloblastoma (n=2), neuroblastoma (n=2) | N/A | N/A | 0 | 0 | no |
| Zorzi 2013 | Pracinostat (SB939) | Alveolar RMS; Embryonal RMS; Ewing sarcoma/peripheral PNET; Lymphoma; Neuroblastoma; Soft tissue sarcoma; Osteosarcoma | N/A, further phases started before P1 study | N/A | 0 | 0 | no |
| Bagatell 2010 | RG1507 | Ewing sarcoma;Rhabdomyosarcoma; CNS tumors; Liver tumors; Osteosarcoma; neuroblastoma, adrenocortical carcinoma (2), Hodgkin's disease, desmoplastic small round cell tumor, epithelioid sarcoma, undifferentiated sarcoma | N/A | N/A | 0 | 0 | no |
| Pearson 2013 | Ridaforolimus | ependymoma (5), osteosarcoma (3), Ewings sarcoma (3) and other histologies (7) | N/A, further phases started before P1 study | N/A | 0 | 0 | no |
| Gore 2013 | Ridaforolimus (MK-8669, AP23573) | sarcoma (osteosarcoma, clear cell sarcoma, chondrosarcoma, Ewing sarcoma, desmoplastic small round cell tumor, soft tissue nonrhabdomyosarcoma, undifferentiated sarcoma), CNS tumors (ependymoma, anaplastic astrocytoma, brainstem glioma, atypical teratoid/rhabdoid tumor), Wilms tumor | N/A, further phases started before P1 study | N/A | 0 | 0 | no |
| Widemann 2012 | Sorafenib | Osteosarcoma, Ewing's sarcoma, Alveolar rhabdomyosarcoma, Synovial sarcoma, Alveolar soft part sarcoma, MPNST, Sarcoma, Hepatoblastoma, Wilms tumor, Adrenocortical carcinoma. Renal cell carcinoma, AML, ALL, Other (NR) | N/A | N/A | 2 | 0 | yes |
| Kieran 2009 | SU5416 (Semaxanib) | Astrocytoma anaplastic; Astrocytoma NOS; Brain stem glioma; Choroid plexus carcinoma; Ependymoma NOS; Ganglioglioma; Glioblastoma multiforme; Glioma malignant; Pilocytic astrocytoma; Pineoblastoma; Primitive neuroectodermal tumor; Rhabdoid sarcoma | N/A | N/A | 0 | 0 | no |
| DuBois 2011 | Sunitinib | Brain tumor; Soft tissue sarcoma; Ewing sarcoma; Neuroblastoma ; Osteosarcoma; Other (desmoplastic small round cell tumor; renal cell carcinoma; spindle epithelial tumor with thymus like differentiation; GIST; malignant meningioma) | N/A | N/A | 2 | 0 | yes |
| Spunt 2011 | Temsirolimus | Rhabdomyosarcoma, Osteosarcoma, Neuroblastoma, Wilms tumor, Germ cell tumor, Adrenocortical carcinoma, Medulloblastoma, Ependymoma, Primitive neuroectodermal tumor, Atypical teratoid rhabdoid tumor Glioblastoma multiforme, Pontine glioma | N/A | N/A | 1 | 0 | yes |
| Coulter 2013 | Temsirolimus, Valproic acid | Alveolar soft part sarcoma, Spinal cord ependymoma grade II, Melanoma, Undifferentiated sarcoma (NOS), Medullary carcinoma thyroid, Hepatocellular carcinoma | N/A | N/A | 0 | 0 | no |
| Widemann 2011 | Tipifarnib | Acute lymphoblastic leukemia (ALL); Acute myeloid leukemia (AML); Chronic myelogenous leukemia (CML); Juvenile myelomonocytic leukemia (JMML) | N/A | N/A | 0 | 1 | yes |
| Leary 2013 | Trebananib (AMG386) | neuroblastoma, rhabdomyosarcoma, Ewing sarcoma, osteosarcoma, other soft tissue sarcoma (NR),nasopharyngeal carcinoma | N/A | N/A | 0 | 0 | no |
| Su 2011 | Valproic acid | Adrenal cortical carcinoma, Atypical teratoid rhabdoid tumor, Brainstem glioma, Mesenchymal chondrosarcoma, Desmoplastic infantile ganglioglioma, Ependymoma, Ewing's sarcoma, Glioblastoma multiforme, Medulloblastoma, Anaplastic oligoastrocytoma, Osteosarcoma, Spindle cell sarcoma, Biphasic synovial sarcoma, Synovial sarcoma NOS, Wilms tumor | N/A | N/A | 0 | 0 | no |
| Fox 2013 | Vandetanib | Medullary thyroid carcinoma | N/A | N/A | 0 | 0 | no |
| Gajjar 2013 | Vismodegib | medulloblastoma | N/A | N/A | 1 | 0 | yes |
| Fouladi 2010a | Vorinostat +/- Retinoic Acid | CNS tumors (Malignant glioma, Medulloblastoma, Astrocytoma, mixed glioma; Ependymoma (NOS); Atypical teratoid/rhaddoid tumor; Primitive neuroectodermal tumor, Pineoblastoma); Non-CNS tumors (Soft tissue sarcomas, Ewing's sarcoma; Neuroblastoma; Osteosarcoma; Rhabdomyosarcoma, Endodermal sinus tumor); Acute lyphoblactic leukemia, Acute promyelocytic leukemia, Acute lmyeloid leukemia FAB M1 | N/A, further phases started later than 5 years since and before P1 study | N/A | 1 | 0 | yes |
