# Supplementary material for: Clinical development success rates and social value of pediatric Phase 1 trials in oncology
Source: PLoS One. 2020 Jun 24;15(6):e0234911. doi: 10.1371/journal.pone.0234911 (PMC7313751; doi:10.1371/journal.pone.0234911)
Supplement: S4 Table — (DOCX) [file pone.0234911.s004.docx]

**S4 Table. Phase 1 transition success, defined as the number of treatments that were advanced to the next pediatric phases according to ClinicalTrials.gov, EU Clinical Trials Register and Google Scholar.**

| **Advanced to the next pediatric phases** | | **ClinicalTrials.gov** | | | **EU Clinical Trials Register** | | | **Google Scholar** | | |
| --- | --- | --- | --- | --- | --- | --- | --- | --- | --- | --- |
|  |  | **yes** | **no** | **p value** | **yes** | **no** | **p value** | **yes** | **no** | **p value** |
|  |  | **number of studies (%)** | |  | **number of studies (%)** | |  | **number of studies (%)** | |  |
| TOTAL | | 18 (12.9) | 121 (87.1) |  | 18 (12.9) | 121 (87.1) |  | 27 (19.4) | 112 (80.6) |  |
| Type of tumor | solid | 14 (13.0) | 94 (87.0) | 0.57 | 11 (10.2) | 97 (89.8) | 0.06 | 17 (15.7) | 91 (84.3) | 0.04 |
|  | hematological | 4 (17.4) | 19 (82.6) |  | 4 (17.4) | 19 (82.6) |  | 6 (26.1) | 17 (73.9) |  |
|  | Both | 0 (0) | 8 (100) |  | 3 (37.5) | 5 (62.5) |  | 4 (50.0) | 4 (50.0) |  |
| Number of drugs | 1 drug | 12 (14.3) | 72 (85.7) | 0.56 | 16 (19.0) | 68 (81.0) | 0.008 | 20 (23.8) | 64 (76.2) | 0.10 |
|  | 2 or more drugs | 6 (10.9) | 49 (89.1) |  | 2 (3.6) | 53 (96.4) |  | 7 (12.7) | 48 (87.3) |  |
| Drug/s generally approved by FDA or EMA before study publication | yes | 12 (13.5) | 77 (86.5) | 0.8 | 15 (16.9) | 74 (83.1) | 0.07 | 22 (24.7) | 67 (75.3) | 0.04 |
|  | no | 6 (12.0) | 44 (88.0) |  | 3 (6.0) | 47 (94.0) |  | 5 (10.0) | 45 (90.0) |  |

p value for differences between type of tumor categories provided by Fisher's exact test

p value for differences between number of drugs and previous approval categories provided by Chi-squared test

percentages show proportion of outcome in each sub-category (e.g. solid tumor)
