# Supplementary material for: Clinical development success rates and social value of pediatric Phase 1 trials in oncology
Source: PLoS One. 2020 Jun 24;15(6):e0234911. doi: 10.1371/journal.pone.0234911 (PMC7313751; doi:10.1371/journal.pone.0234911)
Supplement: S5 Table — (DOCX) [file pone.0234911.s005.docx]

**S5 Table. Number of trials that were cited by primary research reports and systematic reviews**

| **Phase 1 trial cited in primary research reports or practice guidelines** | | **yes** | **no** | **p value** |
| --- | --- | --- | --- | --- |
|  |  | **number of studies (%)** | |  |
| TOTAL | | 130 (94) | 9 (6) |  |
| Type of tumor | solid | 101 (94) | 7 (6) | 0.8 |
|  | hematological | 21 (91) | 2 (9) |  |
|  | Both | 8 (100) | 0 (0) |  |
| Number of drugs | 1 drug | 82 (98) | 2 (2) | 0.029 |
|  | 2 or more drugs | 48 (87) | 7 (13) |  |
| Drug/s generally approved by FDA or EMA before study publication | yes | 82 (92) | 7 (8) | 0.49 |
|  | no | 48 (96) | 2 (4) |  |

p values were calculated using Fisher's exact test

percentages show proportion of outcome in each sub-category (e.g. solid tumor)
