# Supplementary material for: Clinical development success rates and social value of pediatric Phase 1 trials in oncology
Source: PLoS One. 2020 Jun 24;15(6):e0234911. doi: 10.1371/journal.pone.0234911 (PMC7313751; doi:10.1371/journal.pone.0234911)
Supplement: S6 Table — (DOCX) [file pone.0234911.s006.docx]

**S6 Table. Citation patterns of Phase 1 trials**

| Number of citations in Google Scholar | 2060 |
| --- | --- |
| **Type of research** | **number of publications citing Phase 1 study (%)** |
| Pediatric and adult Phase 2 or 3 trials using the same drugs and clinical practice guidelines | 86 (4) |
| Phase 1 trials using the same drugs | 29 (1) |
| Preclinical studies | 848 (41) |
| Trials for other drugs or non-trial biomedical research | 919 (45) |
| Systematic reviews | 178 (9) |
