# Supplementary material for: Clinical development success rates and social value of pediatric Phase 1 trials in oncology
Source: PLoS One. 2020 Jun 24;15(6):e0234911. doi: 10.1371/journal.pone.0234911 (PMC7313751; doi:10.1371/journal.pone.0234911)
Supplement: Attachment — Submitted filename: Final REVIEW TBR.docx [file pone.0234911.s008.docx]

- Title: Clinical development success rates and social value of pediatric phase 1 trials in oncology: A meta-analysis

This paper included a sample of 139 pediatric phase 1 trials in oncology, previously found in a systematic review that retrieved studies from 2004 to 2013 (Waligora et al., 2018), in order to assess:

i. Approval rate (FDA or EMA) - named by the author as translational success,

ii. Rate of advance to further phases (phase 2, 3 or 4) - named by the author as transition success and,

Iii. Citation patterns.

The authors found that 5% of pediatric phase 1 trials in oncology turned to approval in the FDA or EMA, despite a large proportion which have been tested in phase 2, 3 or 4. The trials were widely cited in primary research suggesting a scientific and social impact.

This is a very interesting research topic because pediatric population is vulnerable and few trials have been carried out in this population. Lack of published data increase the off-label use and evidence-based oriented practices is a challenge. United States of America and European Union are the head of a movement to increase pediatric research with special fundings and programs.

The paper aim is very important and open a discussion to understand the barriers to pediatric drug development.

I suggest improvements in the text and the methodology in order to clarify the reader and increase transparency:

1. Title - In my opinion, this paper did a statistical analysis with the data available, not a meta-analysis. A meta-analysis is defined as “a statistical procedure that integrates the results of several independent studies considered to be “combinable” (Egger M, Smith GD, Phillips AN, 1997). I suggest the review of the title;
2. Abstract/Objective - It would be more informative for the reader the definition of ‘translation’ or changing the term ‘translation’ for “approval rate in FDA or EMA”;

Abstract/Methods - Clarify for the reader that the cohort of 139 studies was systematically searched in a previous study (Waligora et al., 2018), in my opinion, it’s important to mention in the Abstract only the methodology of the current paper.

It is unclear to the reader this paragraph “We included small sample size, non‑randomized, dose escalation pediatric cancer phase 1 studies of any malignancy, that assessed chemotherapy and/or targeted therapy and were published between January 1st 2004 and December 31st 2013...”. Is this the inclusion criteria from the previous study? How was it related to other databases searched, FDA and EMA medicine databases, ClinicalTrials.gov and EU Clinical Trials Register or Google Scholar?

If this is the inclusion criteria of the previous systematic review, it would be more clear to maintain it in the beginning of the methodology.

Small sample isn’t an inclusion criteria in the paper methods.

3. Methods

I suggest that the authors clarify what is part of the previous study and what are the methods of the current one. Thus, I suggest that all the information regarding the systematic review, Waligora et al., 2018, remain in the session ‘Study selection and eligibility criteria’ (Pg4/Line73).

Moreover, a critical aspect of this study is the usage of 3 characteristics (‘Type of tumor’, ‘Number of drugs’ and ‘Drug/s generally approved by FDA or EMA before study publication’) in the results and for statistical analysis. However these topics were not mentioned in the methods. The authors should explain the rationale for this 3 characteristics inclusion and its impact in the aim and results of the study.

-Pg4/Line73 - It would be important to mention the databases searched in the previous systematic review (The authors mentioned it in the Abstract).

-Pg4/Line76 - I suggest a Phase 1 definition as their prior paper “Phase I studies, defined as “small sample size, non-randomized, dose escalation studies that defined the recommended dose for subsequent study of a new drug in each schedule tested”” (Waligora et al., 2018)

-Pg4/Line80 - It would be important to reference the pediatric age range, because it can diverge, e.g. the FDA consider pediatrics individuals under 18 years old and the American Academy of Pediatric define pediatrics individuals under 21 years old.

- Pg5/Line96 and Pg5/Line98-104 - Information about previous study and criteria for selection, I suggest to move to ‘Study selection and eligibility criteria’ (Pg4/Line73). How many studies were assessed, 170 or 139? In my understanding all outcomes should use the same timeframe previously specified from 2004 to 2013. The PRISMA flow in the Supplemment material made it clear however, in the text it would be important to fill this gap to the reader understanding.

- Pg5/Line96-98 “After determining the registration….(Table 1) - Table 1 and this paragraph are results. Describe the methods which information the authors will extract from the papers.

-Pg9/153 - I suggest an independent topic about ‘Data extraction’

-Pg9/158-9 - The authors mentioned data extraction related to: “study design, funding, intervention, outcomes and the timing of pediatric testing relative to adult testing as well as data on response and toxicity”. However only funding was reported as result. Please check data extraction.

In the results, it was collected data and performed statistical analysis for ‘Type of tumor’, ‘Number of drugs’ and ‘Drug/s generally approved by FDA or EMA before study publication’ for each outcome (i. Approval rate, ii. Posterior phases rate and iii.Citation pattern), despite it, none of these information were mentioned in the method.

In the PRISMA figure at the Supplemental material and reading Waligora et al., 2018, it’s clear that 139 studies is from 2004 to 2013 and 170 the number retrieved in the previous study from 2004 to 2013.

4. Results

-Pg10/173 - Clarify that ‘One hundred and thirty-nine Phase 1 pediatric oncology trials’ was retrieved from the past study.

- Pg12/209 - In Table 4, I suggest to merge information about Clinicaltrials.gov and EU Clinical Trials Registered. I understood that the analysis aims to access transition to further phases and not compare the protocol-registry databases. Moreover, it’s mandatory the previous registration in the database linked to the trial location, so ClinicalTrials.gov would be mandatory to studies carried out in the USA and EU CT in Europe. Thus, it would be more useful to show the data in a single column, Transition success (advance to further phases in Clinicaltrials.gov **OR** EU Clinical Trials Registered.)

Again, the results and statistical analysis is focusing on three main characteristics not justified for the rational, not mentioned in the methods and not well discussed in the Discussion session.

5. Discussion

Pg12/227 - The authors might describe Phase 1 trials in details, ‘pediatric Phase 1 trials in oncology’.

Pg13/234 - It would be interesting if the author related this increase in single drugs approval with increase in target treatment in oncology that are in the great majority, a single drug. Multiple drugs approval are associated with conventional chemotherapy cytotoxic drugs, which have very few approvals in the past years.

Pg13/242 - This finding isn’t clear in the results.

The discussion is interesting and I additionally suggest that the authors include more discussion about the link between their results and the opportunities and challenges for pediatric research development.
