# Supplementary material for: Clinical development success rates and social value of pediatric Phase 1 trials in oncology
Source: PLoS One. 2020 Jun 24;15(6):e0234911. doi: 10.1371/journal.pone.0234911 (PMC7313751; doi:10.1371/journal.pone.0234911)
Supplement: Attachment — Submitted filename: Response to Reviewers.docx [file pone.0234911.s010.docx]

Dear Editor, Dear Reviewers,

Thank you for accepting our previous revisions. We responses for reviewer’s comments below.

Reviewer #2: Dear all,
The revised manuscript included several alterations and improvements in the methodology and results presentation. The authors addressed many different aspects from the reviewer's comments and added valuable information!
My comments in the previous manuscript were fully responded and I am happy with the alterations.
I have only minor corrections before the publication:

1.pg 5 line91 - In my opinion, this is restricted to the Results session (it is not Methods).
"... We found that 6 drugs tested in 7 trials were approved by at least one agency for pediatric use in oncologic conditions (Supporting information S1 Table, <https://osf.io/36prm/>)"

Response: Thank you for this comment. We move this part to the Results section now.

2.pg 10-11 (Table2) - Please, revise all the values and percentages in Table 2 ("Total", "Number of drugs", "Drug/s generally approved by FDA or EMA before study publication". I calculated and the total was 135, instead of 139.

Response: There was a clarification under the table “Four trials were excluded from the analysis due to not being applicable or an unclear status”. We now added a star to make this information more readable for the future readers.

3. Great job with all supplementary content! In my opinion it might me included in PLOSONE platform as Supplementary material (not only at OSF). Thus, I suggest citation for Supplementary material, not OSF.

Response: Thank you! And many thanks for important suggestions!

Kind regards,

Marcin Waligora
